# Transport and retention of engineered Al_2_O_3_, TiO_2_, and SiO_2_ nanoparticles through various sedimentary rocks

**DOI:** 10.1038/srep14264

**Published:** 2015-09-16

**Authors:** Ali Esfandyari Bayat, Radzuan Junin, Shahaboddin Shamshirband, Wen Tong Chong

**Affiliations:** 1 UTM-MPRC Institute for Oil and Gas, N29A, Lengkuk Suria, Universiti Teknologi Malaysia, 81310, UTM Skudai, Johor Bahru, Malaysia; 2 Department of Computer System and Information Technology, Faculty of Computer System and Information Technology, University of Malaya, 50603 Kuala Lumpur, Malaysia; 3 Department of Mechanical Engineering, Faculty of Engineering, University of Malaya, 50603 Kuala Lumpur, Malaysia

## Abstract

Engineered aluminum oxide (Al_2_O_3_), titanium dioxide (TiO_2_), and silicon dioxide (SiO_2_) nanoparticles (NPs) are utilized in a broad range of applications; causing noticeable quantities of these materials to be released into the environment. Issues of how and where these particles are distributed into the subsurface aquatic environment remain as major challenges for those in environmental engineering. In this study, transport and retention of Al_2_O_3_, TiO_2_, and SiO_2_ NPs through various saturated porous media were investigated. Vertical columns were packed with quartz-sand, limestone, and dolomite grains. The NPs were introduced as a pulse suspended in aqueous solutions and breakthrough curves in the column outlet were generated using an ultraviolet-visible spectrophotometer. It was found that Al_2_O_3_ and TiO_2_ NPs are easily transported through limestone and dolomite porous media whereas NPs recoveries were achieved two times higher than those found in the quartz-sand. The highest and lowest SiO_2_-NPs recoveries were also achieved from the quartz-sand and limestone columns, respectively. The experimental results closely replicated the general trends predicted by the filtration and DLVO calculations. Overall, NPs mobility through a porous medium was found to be strongly dependent on NP surface charge, NP suspension stability against deposition, and porous medium surface charge and roughness.

Nowadays, many industries such as food processing, cosmetics, pigments, paints, and electronics are producing and utilizing engineered metal oxide nanoparticles (NPs) such as aluminum oxide (Al_2_O_3_), titanium dioxide (TiO_2_), and silicon dioxide (SiO_2_)^1^,^2^. This has led to noticeable quantities of these NPs to be released into the environment daily. Since the toxicity of these metal oxide NPs is greater than the toxicity of bulk formulations with the same chemistry^3^, their release and accumulation into the environment has caused adverse influences on aquatic organisms such as microbes, algae, fish, and invertebrates^4^,^5^,^6^. Metal oxide NPs especially Al_2_O_3_ and TiO_2_ have been also classified as carcinogenic to humans by the international agency for research on cancer (IARC)^4^,^7^. A portion of the released NPs can be transferred into the subsurface layers. Since NPs have tiny dimensions, they have the potential to be transported into subsurface alluvial zones^8^,^9^. Thus, there is a high risk that NPs can reach and contaminate drinking groundwater resources. As the physical and chemical properties of subsurface media are extremely complex, issues of how and where the NPs are distributed into the subsurface still remain major challenges for environmental engineering^2^,^10^.

Metal oxide NPs (i.e. Al_2_O_3_, TiO_2_, and SiO_2_) have also been recently introduced as agents for enhanced oil recovery (EOR) from hydrocarbon reservoirs^11^,^12^,^13^. The use of NPs for EOR purposes is a new application in petroleum engineering and needs to be tested and validated before the NPs are fully utilized. NPs usage for EOR is also facing the same question of how NPs are transported through the hydrocarbon reservoirs. NPs can precipitate in hydrocarbon reservoir pores and consequently clog them during transport^14^. Thereby, the permeability of reservoir is reduced which results in a decline of hydrocarbon reservoir productivity. Thus, prior to the use of NPs for EOR implementation, the effects of different parameters on the NPs transport of porous media should be investigated.

As discussed earlier, transport and retention of engineered metal oxide NPs in subsurface natural porous media are important issues for both environmental and petroleum engineering. To date, comprehensive studies on transport and retention of the engineered Al_2_O_3_ and TiO_2_ NPs under some environmentally relevant conditions have been carried out. For example, The effects of different physicochemical parameters such as size, concentration, and shape of NPs^2^,^15^,^16^,^17^,^18^,^19^, NP surface coating with surfactant, polymer and or NOM^20^,^21^,^22^,^23^,^24^, fluid velocity^9^,^15^,^25^, solution chemistry (i.e. ionic strength, pH, and ion type)^21^,^26^,^27^,^28^,^29^,^30^, and presence of clay particles^31^,^32^,^33^ on the transport and retention of the Al_2_O_3_ and TiO_2_ NPs have been determined. However, there are some other parameters that still must to be evaluated. For example, porous media physics and chemistry are parameters which their effects on NPs transport have not yet been fully understood. The earth’s crust is composed of different sedimentary rocks such as sandstones, conglomerates, carbonates, and so on^34^. According to the literature, quartz-sands and soils are two most common types of porous media utilized in previous studies on the NPs transport^2^,^9^,^15^,^21^,^31^,^35^,^36^,^37^. To the best of our knowledge, other porous media types such as carbonate porous media (i.e. limestones and dolomites) have not been fully studied for the NPs transport. Carbonates are a class of sedimentary rocks which form about 15% of the earth’s sedimentary crust^34^. A considerable portion of underground water resources is located within the carbonate rocks^29^. In addition, approximately 40% of the discovered hydrocarbon resources in the world is in the carbonate rocks^38^,^39^. Thus, more studies are in demand to evaluate the NPs transport behavior through carbonate rocks. Consequently, the role of porous media physics and chemistry on NPs transport will be figured out.

This study was designed to investigate and compare the influences of three sedimentary rocks (i.e. limestone, dolomite, and quartz-sand) on transport and retention behavior of three metal oxide NPs, namely Al_2_O_3_, TiO_2_, and SiO_2_. To achieve this aim, laboratory scale column experiments were carried out to determine the amount of Al_2_O_3_, TiO_2_, and SiO_2_ NPs retention in limestone, dolomite, and quartz sand porous media. Tests included ultraviolet-visible (UV-VIS) spectrophotometer, dynamic light scattering (DLS) and zeta potential (ζ-potential) techniques which were performed to measure the NP concentration, size and surface charge, respectively. Furthermore, the classical Derjaguin–Landau–Verwey–Overbeek (DLVO) and filtration theories were applied to assess and explain the NPs deposition and maximum transport distance through porous media.

## Results and Discussion

### Nanoparticles Characterizations

The average size of Al_2_O_3_, TiO_2_, and SiO_2_ nanopowders was measured by two methods. Firstly, X-ray diffraction (XRD) analysis and Scherrer’s formula^40^ were used as follows:


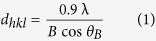


where *d*_*hkl*_ is the mean NP size (nm), *B* (radians) comprises the full width at half-maximum of the broadened diffraction line observed at the 2θ angular range, λ is the wavelength of Cu K_α_ radiation (λ = 0.1542 nm) and *θ*_*B*_ is the Bragg angle of diffraction.

**Figure 1:**
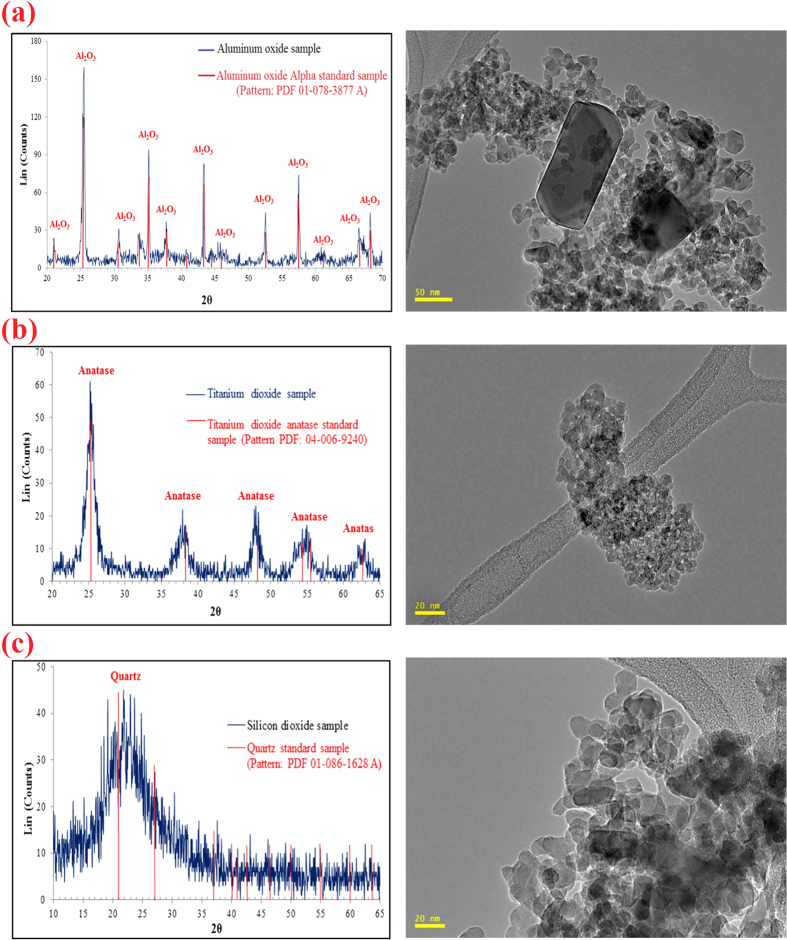
XRD (left) and TEM (right) analyses from the nanopowders (**a**) Al_2_O_3_-NPs, (**b**) TiO_2_-NPs, (**c**) SiO_2_-NPs.

The XRD analysis from aluminum oxide sample demonstrated that NP has alpha (α) crystalline structure and its composition is pure Al_2_O_3_ (Fig. 1a). TiO_2_ and SiO_2_ NPs have partial amorphous (semi-crystalline) structures. The former has a composition of anatase while the latter has quartz composition as shown in Fig. 1b,c. Furthermore, from the equation, the sizes of Al_2_O_3_, TiO_2_, and SiO_2_ NPs were calculated and found to be 26, 11.8, and 16.1 nm respectively. The sizes of Al_2_O_3_, TiO_2_, and SiO_2_ nanopowders were also measured by TEM and image-processing software (Image*J*; National Institute of Mental Health). The geometric means of Al_2_O_3_, TiO_2_, and SiO_2_ NPs diameter measured were 25 (12–138), 6 (3–65), and 13 (7–81) nm respectively. Therefore, there is a 35% to 40% difference in the size of the NPs depending on whether it was measured by XRD and TEM or reported by the manufacturer. Furthermore, according to TEM images, the morphology of NPs appeared to be spherical (Fig. 1).

The ζ-potential values of Al_2_O_3_, TiO_2_ and SiO_2_ NPs in DIW were also measured to be +19.1 ± 0.3, +9.1 ± 0.3, and −28.1 ± 0.3 mV respectively. Besides that, the sizes of Al_2_O_3_, TiO_2_, and SiO_2_ NP aggregates in DIW were measured to be 255 (75–510), 220 (65–440), and 280 (80–620) nm respectively using the DLS probe. The DLS analyses depict that the hydraulic size of Al_2_O_3_, TiO_2_, and SiO_2_ NPs in DIW is much greater than the size of NPs in the powder form. Thus, it can be concluded that these NPs aggregated in DIW.

### Sedimentation tests results

**Figure 2:**
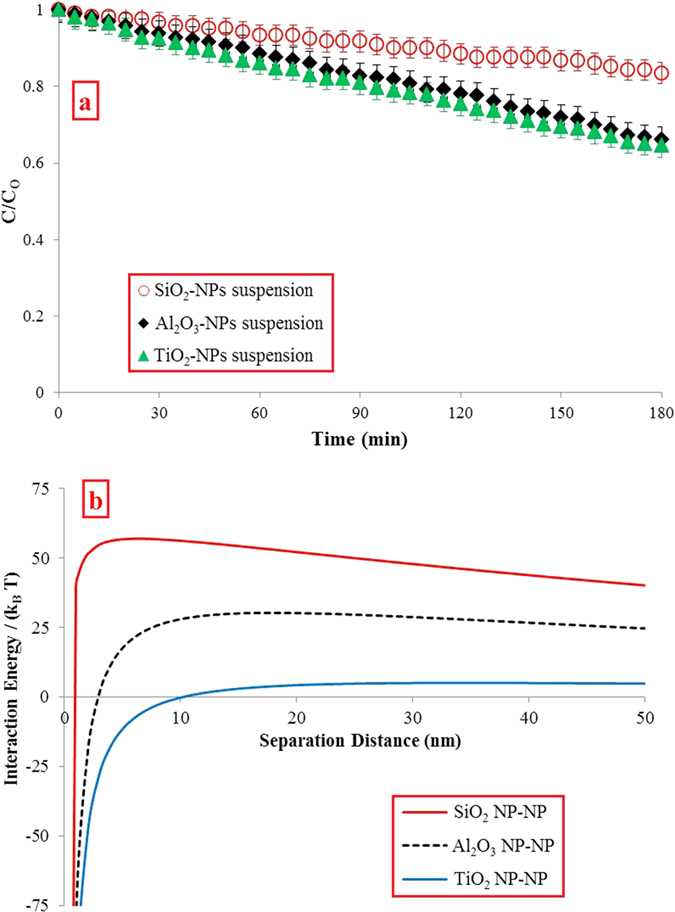
(**a**) Sedimentation tests results, (**b**) NP-NP interaction energy profiles generated by DLVO theory.

The sedimentation tests results revealed that the stability of SiO_2_-NPs against deposition in static condition was higher than Al_2_O_3_ and TiO_2_ NPs during 180 min (Fig. 2a). The interaction between NPs is one of the factors which determines the NPs stability in a dispersion medium^29^. For this finding, DLVO theory was applied to qualitatively explain this interaction^41^. The interaction between two particles can either be attraction or repulsion. The electrostatic double layer (EDL) is a force which drives particles apart while the van der Waals (VDW) force drives particles toward each other. The summation of these forces clarifies whether the net interaction between two particles is attractive or repulsive^42^. High positive values of the total interaction energy imply that the EDL force is greater in magnitude and the dominant force^29^. The total interaction energy for NP-NP as a function of distance for the Al_2_O_3_, TiO_2_, and SiO_2_ NPs was calculated and drawn. As shown in Fig. 2b, the EDL is the dominant force among all NPs. However, the interaction energy height for SiO_2_-NPs is higher than Al_2_O_3_ and TiO_2_ NPs which reveals a higher repulsion force among SiO_2_-NPs. Consequently, SiO_2_-NP suspension is more stable than Al_2_O_3_ and TiO_2_ NPs suspensions. Therefore, DLVO theory supports these experimental results.

It should be pointed that for applying filtration theory, the condition should be steady state during the transport tests^21^. According to 1 ml/min injection rate and number of injected PVs (2PVs) during the experiments, 18 min is required to deliver the NPs suspensions into the porous media. Based on the optical absorbency results (Fig. 2a), for the first 20 min of the experiments, NPs sedimentation values are less than 5 wt% of the initial value (0.005 wt%) that is negligible. Thus, it is justifiable to apply the transport theory for steady-state systems.

### Nanoparticles transport through quartz-Sand porous medium

**Figure 3:**
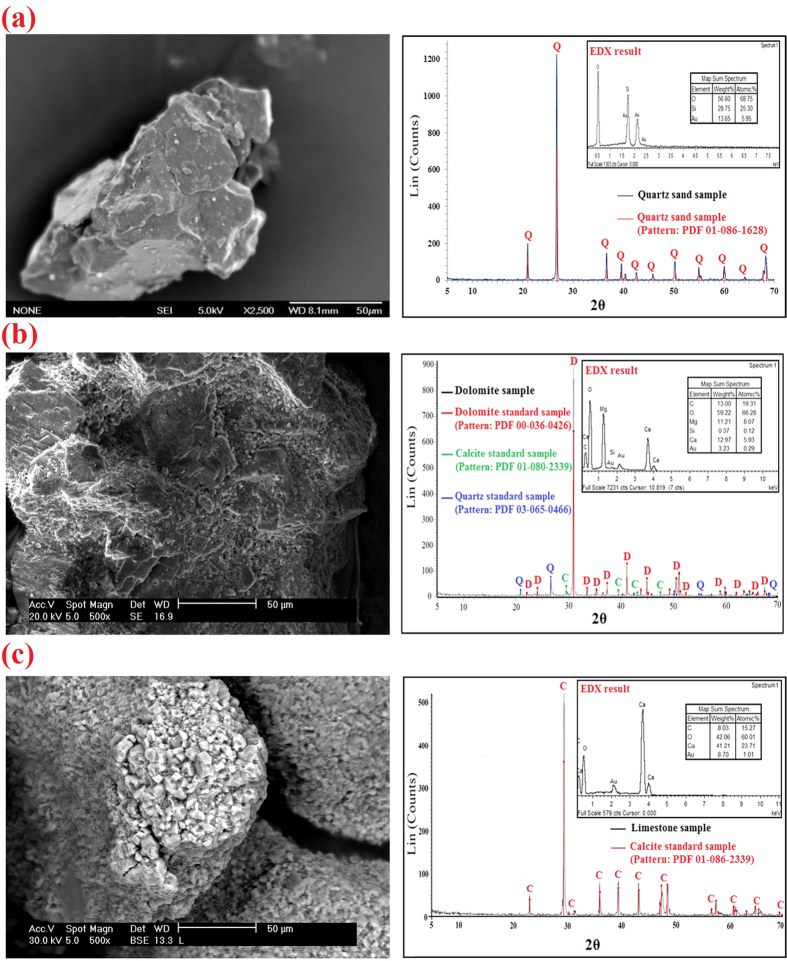
SEM, XRD, and EDX analyses results from porous media (**a**) quartz sand, (**b**) dolomite, (**c**) limestone.

Different characterizations were carried out on the quartz-sand sample before the column experiments. The SEM analysis from quartz-sand grains depicted that the surface of grains is smooth (Fig. 3a). Furthermore, the XRD and EDX analyses from the quartz sample also revealed that the main mineral in the sample is quartz (SiO_2_) without any impurities (Fig. 3a). Besides that, the quartz-sand surface charge was measured as −36.2 ± 0.5 mV.

**Figure 4:**
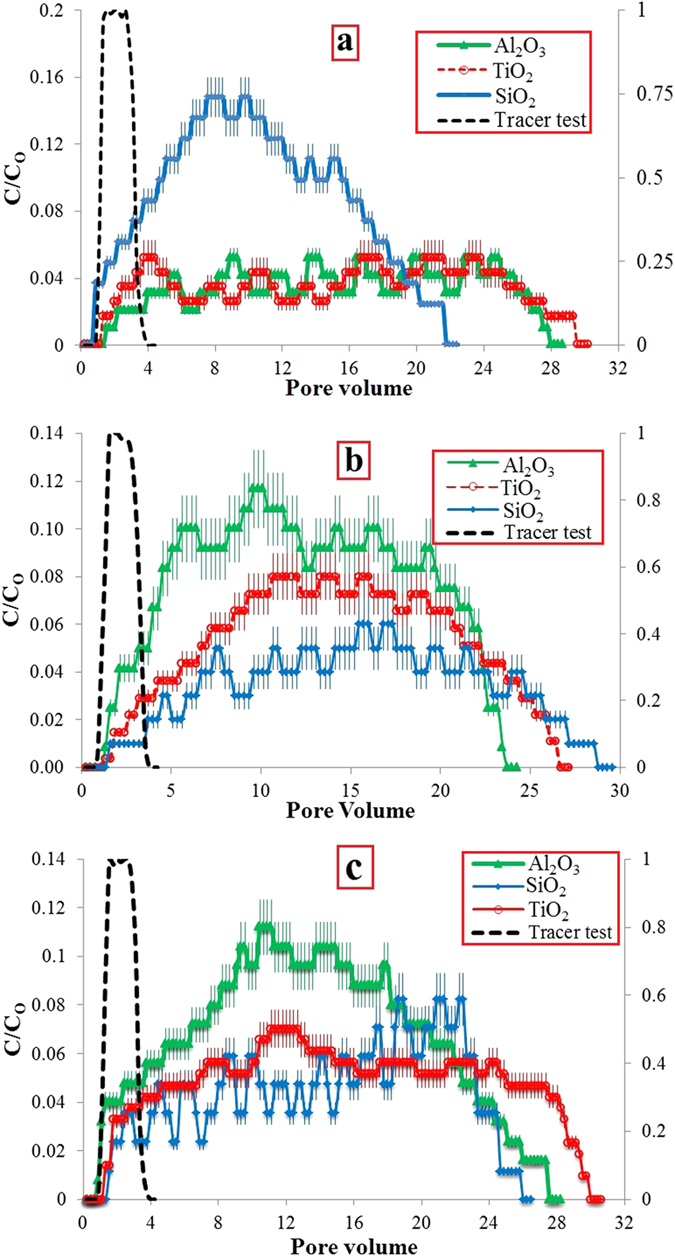
NPs breakthrough curves transported through various porous media; (**a**) quartz sand, (**b**) dolomite, (**c**) limestone. Breakthrough curve for the tracer test is also demonstrated; Right y-axis for tracer (KNO_3_) C/C_O_ and left y-axis is for NPs C/C_O_.

Once the columns were packed, their porosities (*φ*) and permeability coefficients (*k*) were firstly measured. The average *φ* and *k* of the packed columns were measured as 43% and 2.96 × 10^−12^ m^2^ respectively. The tracer tests demonstrated that the flow path was open and the injected KNO_3_ solution was eluted entirely after injection of 2 PVs of DIW (Fig. 4a). Thereafter, the NPs transport tests were carried out. The first transport test was carried out by Al_2_O_3_-NP suspension. Presence of Al_2_O_3_-NPs was observed in the outlet after 1.4 PVs injection. The NP concentration in the effluent moderately increased until 5.0 PVs. Then, the NP concentration (C/C_O_) values fluctuated around 0.04 until the 25^th^ PVs. Thereafter, the NPs concentration declined to zero at the 28^th^ PVs. After that, Al_2_O_3_-NPs were not observed in the outlet even after addition of extra 10 PVs of DIW. The result demonstrated that 47.6% of the entered Al_2_O_3_-NPs were recovered from the column. The NP breakthrough curve is demonstrated in Fig. 4a.

**Figure 5:**
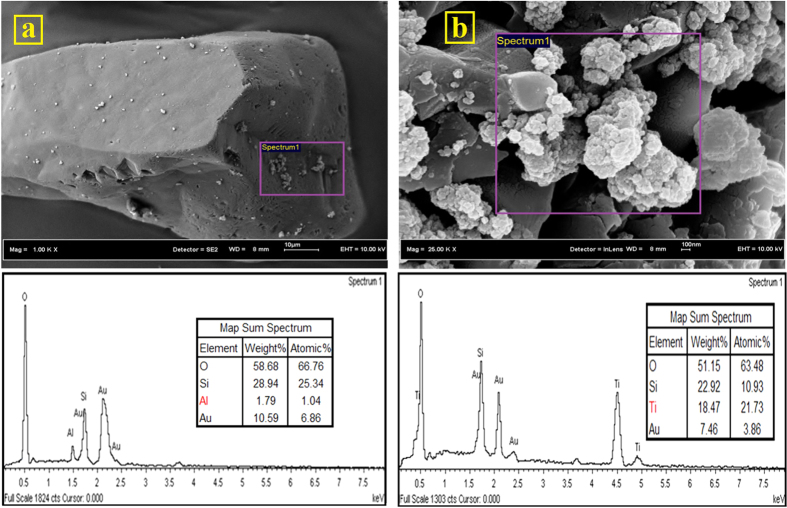
(**a**) FESEM-EDX analyses from quartz sand grains after the transport experiments, (**a**) Al_2_O_3_-NPs, (**b**) TiO_2_-NPs. (The images were taken from grains located in the entrance of columns).

The surface charge of NPs is a primary factor in their transport through porous media^43^. The low Al_2_O_3_-NP recovery from the column is attributed to the huge difference between electric surface charges of Al_2_O_3_-NPs (+19.1 ± 0.3 mV) and quartz-sand grains (−36.2 ± 0.5 mV). According to DLVO theory (Figure S1a), the electrostatic barrier energy height between Al_2_O_3_-NPs and quartz-sand grains is negative indicating that there is an attraction force (VDW) between NPs and quartz-sand grains. As a result of this attraction force, the affinity of Al_2_O_3_-NPs to attach to the quartz-sand grains surfaces is high. Thus, the attachment process is the key retention mechanism for Al_2_O_3_-NPs in the quartz-sand porous medium. The attachment of Al_2_O_3_-NPs on the quartz-sand grains surfaces was also confirmed by FESEM and EDX analyses. Figure 5a shows that Al_2_O_3_-NPs are widely attached on the surfaces of quartz-sand grains. The values of Al_2_O_3_-NPs attachment efficiency, maximum transport distance, and deposition coefficient rate, calculated from filtration theory are shown in Table 1. Darlington *et al.*^2^ also found that Al_2_O_3_-NPs are strongly attached to the soil and sand grains surfaces.

The second transport test using TiO_2_-NPs. through quartz-sand porous media was carried out. The NPs appeared in the outlet after 1.2 PVs injection. The TiO_2_-NPs breakthrough curve is shown in Fig. 4a. The result revealed that 51.7% of the influent TiO_2_-NPs was recovered from the column. A low TiO_2_ NP recovery is due to the difference in electric surface charge signs of TiO_2_-NPs (+9.1 ± 0.3 mV) and quartz-sand grains (−36.2 ± 0.5 mV). Dunphy Guzman *et al.*^43^ declared that surface charge of TiO_2_-NPs is a primary factor in their transport through soils. Choy *et al.*^44^ and Godinez and Darnault^21^ observed that TiO_2_-NPs adsorbed highly on sand grains surfaces due to their opposite surface charge signs. Moreover, Chen *et al.*^28^ reported a successful transport of TiO_2_-NPs through sand grains. They reported that TiO_2_-NPs and sand grains had similar surface charge signs as both were reported to be negative.

According to DLVO theory, the electrostatic barrier energy height between TiO_2_-NPs and quartz grains is negative which reveals attraction is the dominant force (Figure S1a). Therefore, the attachment process is the key retention mechanism for TiO_2_-NPs in quartz-sand porous media. The NPs attachment to the grains surfaces was also proven via FESEM and EDX analyses (Fig. 5b). The values of attachment efficiency, maximum transport distance, and deposition coefficient rate calculated by filtration theory were 0.171, 37.3 cm, and 4.72 (h^−1^), respectively (Table 1).

Transport of SiO_2_-NPs through quartz-sand porous medium was the last test. SiO_2_-NPs were observed in the outlet immediately after injecting the first PV (Fig. 4a). Concentration of SiO_2_-NPs in the effluent sharply increased to 0.148 in the 9.8^th^ PV. Thereafter, it temperately dropped to zero at 21.8^th^ PV. 95.6% of the entered SiO_2_-NPs was collected from the column. The high SiO_2_-NP recovery is attributed to its stability in DIW as well as the electric surface charge of SiO_2_-NPs (−28.1 ± 0.3 mV) and quartz-sand grains (−36.2 ± 0.5 mV) which are both negative. According to DLVO theory, there is a relatively strong electrostatic barrier energy between SiO_2_-NPs and quartz-sand grains which indicates a strong repulsion (EDL) force between them. As a result of this repulsion force, the affinity of SiO_2_-NPs to attach to the sand grains surfaces was quite low.

### Nanoparticles transport through dolomite porous medium

Based on SEM analysis, the surface of dolomite grains was detected to be rough and full of irregular dents and bumps (Fig. 3b). Moreover, the XRD and EDX results revealed that the sample is composed mainly of calcium magnesium carbonate, CaMg(CO_3_)_2_. Small amounts of quartz were also detected in this sample (Fig. 3b). Besides that, the dolomite surface charge was measured as +26.1 ± 0.5 mV.

The average *φ* and *k* of the packed columns were measured as 42% and 3.19 × 10^−12^ m^2^ respectively. The tracer tests demonstrated that the flow path is open and the injected KNO_3_ solution was eluted entirely after injection of 2 PVs of DIW.

The first transport test was run by Al_2_O_3_-NP suspension. The Al_2_O_3_-NPs breakthrough curve is shown in Fig. 4b. The result demonstrated that 89.6% of injected Al_2_O_3_-NPs was recovered from the column. The high Al_2_O_3_-NP recovery is attributed to the electric surface charge signs of Al_2_O_3_-NPs (+19.1 ± 0.3 mV) and dolomite grains (+26.1 ± 0.5 mV) which are both positive. Based on DLVO theory, there is a strong electrostatic barrier energy between Al_2_O_3_-NPs and dolomite grains which depicts a strong repulsion force between Al_2_O_3_-NPs and grains (Figure S1b). As a result of this repulsion force, the affinity of Al_2_O_3_-NPs to attach to the dolomite grains surfaces was low. However, a small amount of Al_2_O_3_-NPs (10.4%) remained in the porous medium and this can be attributed to the roughness of the surface of dolomite grains and presence of small amounts of quartz in the sample. According to filtration theory, the values of attachment efficiency, maximum transport distance, and deposition coefficient rate, for Al_2_O_3_-NP transport through dolomite porous medium, were calculated to be 0.134, 44.5 cm and 4.14 (h^−1^) respectively (Table 1).

**Figure 6:**
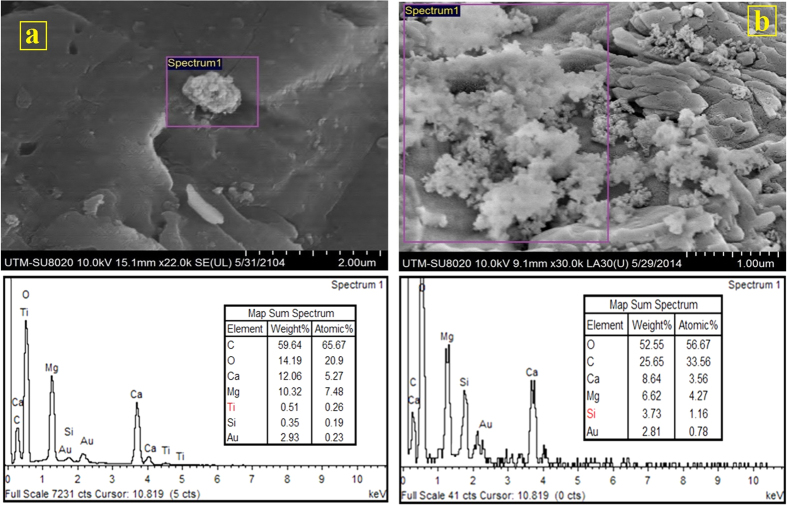
(**a**) FESEM-EDX analyses from dolomite grains after the transport experiments, (**a**) TiO_2_-NPs, (**b**) SiO_2_-NPs. (Right image was taken from a grain located in the top of column and left image was taken from a grain located in the entrance of column).

The next transport experiment was performed by TiO_2_-NP suspension. The result depicted that 68.8% of TiO_2_-NPs influent was recovered from the column. The surface charges of TiO_2_ (+9.1 ± 0.3 mV) and dolomite grains (+26.1 ± 0.5 mV) are both positive. According to DLVO, the interaction energy barrier is positive which depicts that a repulsion force exists between TiO_2_-NPs and dolomite grains. However, one third of the entered TiO_2_-NPs remained in the porous medium. To know why the NPs retained in the porous medium, the results from this test should be compared with Al_2_O_3_-NPs transport test. According to DLVO, the maximum interaction energy (k_B_T) height value for TiO_2_ NP-NP is one sixth of the interaction energy height for Al_2_O_3_ NP-NP (Table 1) which shows stronger repulsion forces among Al_2_O_3_-NPs. Thus, the stability of Al_2_O_3_-NPs in DIW against deposition is higher than TiO_2_-NPs. Yu *et al.*^14^ and Bayat *et al.*^29^,^30^ stated that mobility of NPs through a porous medium is a function of their stabilities where lower stabilities lead to mobility reduction. In addition, the interaction energy height between Al_2_O_3_-NPs and dolomite grains is 4.4 folds higher than TiO_2_-NPs and dolomite grains. This depicts that the repulsion force between Al_2_O_3_-NPs and dolomite grains is much greater than TiO_2_-NPs and dolomite grains (Table 1). In addition, according to filtration theory, the values of attachment efficiency and deposition rate coefficient for TiO_2_-NPs in dolomite porous media are also 1.25 times higher than Al_2_O_3_-NPs as shown in Table 1. The value of attachment efficiency has direct relationship with the amount of NP recovery where higher values of attachment efficiency lead to lower amounts of NPs recovery from the column^21^,^30^,^31^,^45^. These are the reasons why Al_2_O_3_-NPs recovery value in dolomite porous media is higher in comparison to TiO_2_-NPs. Consequently, deposition process is the key retention mechanism for TiO_2_-NPs in the dolomite porous medium. Results of the FESEM and EDX analyses show that the deposited TiO_2_-NPs were also observed on the grains surfaces located in the middle of column (Fig. 6a).

SiO_2_-NPs transport was the last test through dolomite porous medium. The outcome revealed that only 48.6% of the entered SiO_2_-NPs in the column was recovered. Difference in the electric surface charge signs of SiO_2_-NPs (−28.1 ± 0.3 mV) and dolomite grains (+26.1 ± 0.5 mV) caused the NPs to attach strongly to the dolomite grains surfaces. The result of DLVO theory demonstrates that there is an attraction force between SiO_2_-NPs and sand grains (Figure S1b). Consequently, FESEM and EDX measurements were carried out to observe the attached NPs. As shown in Fig. 6b, SiO_2_-NPs were widely attached to the dolomite grains surfaces. As mentioned in Nanoparticles characterizations section, the NPs in DIW aggregated and made bigger clusters. The bigger NPs clusters during transport through porous media can trap behind the tiny pore-throats which this phenomenon is called straining. The trapped clusters may then make networks up to micron size especially when the NPs and porous media grains surface charges are in contrast. Therefore, the NPs aggregates observed in the some FESEM images. Furthermore, based on filtration theory, the values of attachment efficiency, maximum transport distance, and deposition coefficient rate were calculated to be 0.203, 33 cm and 5.33 h^−1^ respectively (Table 1).

### Nanoparticles transport through limestone porous medium

From the SEM analysis, the surface of limestone grains was detected to be rough and full of irregular dents and bumps which are similar to dolomite grains (Fig. 3c). Furthermore, the XRD and EDX results revealed that the sample is composed purely of calcite, CaCO_3_. The limestone surface charge was also measured as +33.1 ± 0.5 mV.

**Figure 7:**
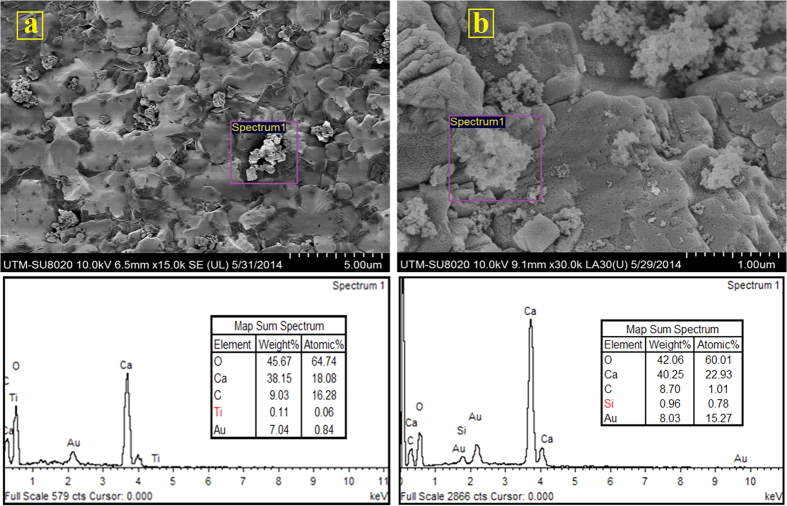
(**a**) FESEM-EDX analyses from limestone grains after the transport experiments, (**a**) TiO_2_-NPs, (**b**) SiO_2_-NPs. (Right image was taken from a grain located in the top of column and left image was taken from a grain located in the entrance of column).

The average *φ* and *k* of the packed columns were measured as 44% and 3.05 × 10^−12^ m^2^ respectively. The tracer tests results depicted that the flow path is open (Fig. 4c). The breakthrough curves for Al_2_O_3_, TiO_2_, and SiO_2_ NPs transport tests through limestone porous media are also shown in Fig. 4c. The results demonstrated that 91.8% of injected Al_2_O_3_-NPs, 72.2% of TiO_2_-NPs, and 56.6% of SiO_2_-NPs were recovered from the columns. The interaction energy curves between NPs and limestone grains were drawn based on DLVO theory. The maximum and minimum interaction energy heights belong to Al_2_O_3_ and SiO_2_ NPs (Table 1). The reasons for the high and/or low NPs recovery have been explained in the previous sections. Deposition and attachment respectively were detected as the key retention mechanisms for TiO_2_ and SiO_2_ NPs on limestone grains. Accordingly, the FESEM and EDX measurements proved the presence of TiO_2_ and SiO_2_ NPs on the limestone grains surfaces (Fig. 7). In addition, based on filtration theory, the maximum values for the both attachment efficiency and deposition rate coefficient parameters in the limestone porous media were obtained for SiO_2_-NPs while the minimum values of the mentioned parameters were achieved for Al_2_O_3_-NPs (Table 1).

The outcomes from this study are of interest to those in the environmental and petroleum engineering fields. Generally, NPs transport and retention through various porous media was found to be strongly dependent on NP stability in suspension against deposition, NP surface charge as well as porous media surface charge and roughness. The more stable NPs which have the same surface charge sign as porous media can easily transport while contrast in surface charge sign between NPs and porous media leads to NPs to attach to the porous media grains surfaces. Consequently, the quantities of Al_2_O_3_, TiO_2_, and SiO_2_ NPs recoveries from quartz-sand porous media were totally different from the carbonates (limestone and dolomite) porous media. For Al_2_O_3_ and TiO_2_ NPs, attachment process is the key retention mechanism through the quartz-sand porous medium. Moreover, it was observed that TiO_2_-NPs recoveries through the carbonates porous media were lower than Al_2_O_3_-NPs recoveries. The reason is related to the lower stability of TiO_2_-NPs in DIW as compared to Al_2_O_3_-NPs. Deposition and attachment processes have been identified as key retention mechanisms for TiO_2_-NPs and SiO_2_-NPs in the carbonates porous media.

## Methods

### NPs suspension preparation and characterization

Three commercial nanopowders, SiO_2_ (20 nm, non-porous), Al_2_O_3_ (40 nm, alpha-Al_2_O_3_), and TiO_2_ (10–30 nm, anatase-TiO_2_) received from SkySpring Nanomaterials, Inc., (Houston, TX), were utilized in this study. X-ray diffraction (XRD, model D5000, SIMENS) and transmission electron microscopy (TEM, model JEM-2100/HR, JEOL, Acc.200.00 kV) analyses were carried out to recheck the NPs crystalline compositions, sizes, and morphologies. Al_2_O_3_, TiO_2_, and SiO_2_ NPs suspensions were prepared by adding 50 mg of each nano powder to 1 liter of de-ionized water (DIW) at pH 6.4 ± 0.1. The reason for selecting DIW as the dispersion medium was to avoid the effects of solution chemistry (i.e. ionic strength and pH) on the NPs stability and transport. The suspensions were agitated for 1 hr using an orbital shaker at 220 rpm and ultrasonicated by an ultrasonic bath for a period of 1 hr to obtain homogenous suspensions prior to each test. The NPs suspensions preparation was carried out 15 min prior to the tests.

For the experiments, the average ζ-potential values of the NPs in DIW were measured via a zeta potential analyzer instrument (ZEECOM Microtec Co., Ltd). The NPs suspensions were prepared, sonicated for 1 hr and shaken prior used for the ζ-potential measurements. The ζ-potential values were obtained by averaging three ζ-potential measurements for all NPs suspensions. Furthermore, the average NP aggregates radiuses in DIW were measured using a DynaPro Titan Dynamic Light Scattering (DLS) probe from Wyatt Technology Corporation. Then, the NPs suspensions were prepared for ζ-potential measurements. DLS scattering analyses were performed thrice (20 DLS reading per each run) for each NP suspension. The mean value of the measurements was applied to determine the radius of NPs aggregates. All these characterization techniques were conducted at room temperature (26 °C).

### Sedimentation tests

The stability of Al_2_O_3_, TiO_2_, and SiO_2_ NPs in DIW against deposition was determined through sedimentation experiments. The sedimentation tests were carried out in the static condition where there was no movement in the suspensions. The amount of NPs deposition was recorded for each 5 min intervals over a 180 min time span with the use of time-resolved optical absorbance. The absorbance of the samples was measured using an ultraviolent visible (UV-VIS) spectrophotometer (model 105, BUCK SCIENTIFIC., Inc.) over the wavelength range of 200–800 nm. Calibration was based on the maximum absorbance wavelength of 400 nm. These experiments were repeated twice and the presented data is the average of the obtained records.

### Porous media preparation and characterization

Natural limestone (from a surface outcrop in Ipoh, Malaysia), dolomite (from Ward’s Natural Science Establishment, Inc., Minnesota, NY, US), and quartz-sand (from Desaru seaside, Johor Malaysia) were utilized as the porous media used in this study. The samples of limestone and dolomite were received as large blocks. The blocks were broken off into the smaller pieces and then crushed to fine grains using a crusher machine (PULVERIZER Type from BICO, Inc.,). The limestone, dolomite, and quartz-sand grains were sifted through 125 and 175 μm stainless steel sieves (USA Standard Testing Sieves, ATM Corp., New Berlin, WI) to achieve an average collector diameter (d_c_) of 150 μm. The limestone and dolomite fractions were pre-treated using a sequential water rinse, ultrasonication, and oven-drying procedure at 110 °C for 12 hours to eliminate impurities. The quartz-sand grains were also cleaned from impurities by soaking them in 12 N HCL acid (Fisher Scientific) for 24 hours, washing in de-ionized water (DIW), and baking at 250 °C for 12 hours^28^. The densities of limestone, dolomite, and quartz-sand samples after pre-treatment were measured according to the ASTM standard D 854–14 method^46^ as 2.67, 2.83, and 2.52 g/cm^3^ respectively. Following that, scanning electron microscope (SEM, Philips XL40) and field emission scanning electron microscopy (FESEM, HITACHI, SU8020) images were prepared from the limestone, dolomite, and quartz-sand grains to determine the exact morphology of the grains before and after performing the transport experiments. Besides that, energy dispersive X-ray (EDX) and XRD analyses were also carried out to determine the limestone, dolomite and quartz-sand compositions before and after the transport tests. The ζ-potential values of the cleaned samples were also measured using the method detailed by Tufenkj and Elimelech^45^.

### Column transport tests

For each experimental trial, the cleaned limestone, dolomite, and quartz-sand grains averaging 30.8, 32.8, and 29.3 gr respectively were placed into stainless steel tube with an inner diameter of 0.9 cm and a length of 33 cm. A 50 micron filter cloth was placed at both ends of the tube to prevent grain migration during displacement tests. The column was then wet packed uniformly with cleaned grains using the method explained by Chen *et al.*^28^. Once the column was packed, it was saturated in a vertical upward direction using a syringe pump (Model PSK-01; NIKKISO Co., Ltd) at a constant Darcy velocity of 4.66 × 10^−3^ cm/sec (flow rate of 1 cm^3^/min) with approximately 10 pore volumes (PVs) of DIW to ensure a homogenous saturation of the pack. It should be noted that the flow direction was selected to be vertically upward to equilibrate the influent solution and enhance packing homogeneity^21^. Once the column was homogeneously packed and saturated, a pulse of each NP suspension (50 mg/L) with Darcy velocity of 4.66 × 10^−3^ cm/sec was injected into the column for 2 PVs. Thereafter, DIW was injected into the column until no NP was observed in the outlet as a means to check for mobilization of trapped NPs in the porous medium. At the same time, the effluents were collected using a fraction collector (CF-2, Spectrum Chromatography, Houston, TX, USA) in 2 ml sample sizes. On average, 170 effluent samples were collected after each conducted experiment. The NPs concentration in the collected samples was measured using an UV-VIS spectrophotometer (Model 105, BUCK SCIENTIFIC., Inc.) over the wavelength range of 200–800 nm. Calibration was based on a maximum absorbance wavelength of 400 nm. Finally, the concentration of NPs dispersions entering the porous medium C_O_, and in the outlet C were applied to generate breakthrough curves of C/C_O_ as function of PVs passing through the porous media. All transport experiments were duplicated and data expressed is the standard error of mean.

Prior to transport tests, a tracer test was also carried out using a 50 mM potassium nitrate (KNO_3_) solution to determine the water flow characteristics and column performance. The selection of KNO_3_ is related to its stability and non-reactive species^28^. 2 PVs of KNO_3_ were injected into the porous media. Consequently, DIW was injected and the outlets were collected in 2 ml samples. The concentration of KNO_3_ in the outlet was measured using a conductivity probe (Thermo Scientific Orion 3-Star). Finally, the concentration of KNO_3_ entering the column C_O_, and in the outlet C was applied to generate breakthrough curves of C/C_O_ as function of PVs passing through the column.

### Colloids transport theories

The transport tests results were cross checked by classical DLVO and filtration theories. These theories were applied to qualitatively and quantitatively asses and explain the NPs transport and retention through porous media^29^,^30^. The details of these theories are illustrated in the supplementary file.

## Additional Information

**How to cite this article**: Esfandyari Bayat, A. *et al.* Transport and retention of engineered Al_2_O_3_, TiO_2_, and SiO_2_ nanoparticles through various sedimentary rocks. *Sci. Rep.* **5**, 14264; doi: 10.1038/srep14264 (2015).

## Supplementary Material

Supplementary Information

## Figures and Tables

**Table 1:** Electrokinetic properties of NPs and porous media, energy barrier heights as calculated by DLVO theory for NP-NP and NP-porous medium interactions as well as experimental parameters from NPs transport tests calculated by filtration theory.

**Porous media**	**NP suspension**	**DLVO Theory**				**Filtration Theory**				R_NP_%
		**NPs ζ-potential (mV)**	**Maximum value of NP-NP interaction energy height**	**Porous media ζ-potential (mV)**	**Maximum value of NP- medium interaction energy height**	**Single-collector contact efficiency,** ***η***_***0***_*** = η***_***D***_*** + η***_***I***_*** + η***_***G***_	**Attachment efficiency, α**	**Maximum transport distance, L**_**max**_ **(cm)**	**Deposition rate coefficient, k**_**d**_ **(h**^**−1**^)	
Quartz sand	Al_2_O_3_	+19.1 ± 0.3	+30.2	−36.2 ± 0.5	negative	1.34E-02	0.182	33.0	5.46	47.6
	TiO_2_	+9.1 ± 0.3	+5.2		negative	1.30E-02	0.169	37.3	4.72	51.7
	SiO_2_	−28.1 ± 0.3	+57.1		+194.5	1.19E-02	0.132	50.4	3.66	95.6
Dolomite	Al_2_O_3_	+19.1 ± 0.3	+30.2	+26.1 ± 0.5	+82.1	1.33E-02	0.134	44.5	4.14	89.6
	TiO_2_	+9.1 ± 0.3	+5.2		+18.6	1.25E-02	0.168	37.3	5.06	68.8
	SiO_2_	−28.1 ± 0.3	+57.1		negative	1.23E-02	0.203	33.0	5.33	48.6
Limestone	Al_2_O_3_	+19.1 ± 0.3	+30.2	+33.1 ± 0.5	+88.3	1.35E-02	0.127	47.2	3.81	91.8
	TiO_2_	+9.1 ± 0.3	+5.2		+19.1	1.26E-02	0.143	44.2	4.18	72.2
	SiO_2_	−28.1 ± 0.3	+57.1		negative	1.24E-02	0.168	40.3	4.27	56.6
