# Supplementary material for: Transport and retention of engineered Al2O3, TiO2, and SiO2 nanoparticles through various sedimentary rocks
Source: Sci Rep. 2015 Sep 16;5:14264. doi: 10.1038/srep14264 (PMC4571619; doi:10.1038/srep14264)
Supplement: Supplementary Information [file srep14264-s1.pdf]

Supplementary Information

Transport and retention of engineered Al_2O_3, TiO_2, and SiO_2 nanoparticles through various sedimentary rocks

Ali Esfandyari Bayat[1]*; Radzuan Junin[1]; Shahaboddin Shamshirband[2]; Chong Wen Tong[3]

[1]UTM-MPRC Institute for Oil and Gas, N29A, Lengkuk Suria, Universiti Teknologi Malaysia, 81310, UTM Skudai, Johor Bahru, Malaysia
[2]Department of Computer System and Information Technology, Faculty of Computer System and Information Technology, University of Malaya, 50603 Kuala Lumpur, Malaysia
[3]Department of Mechanical Engineering, Faculty of Engineering, University of Malaya, 50603 Kuala Lumpur, Malaysia

***Corresponding Author:** Ali Esfandyari Bayat

Email: *ali.esfandiari.bayat@gmail.com*; **HP:**(+60) 147 152 595

1. Introduction

The purpose of this file is to provide additional information of interest to the readers. The colloids transport theories include filtration and DLVO are illustrated in detail in this section.

2. *Filtration Theory*

Filtration theory is mainly applied to quantitatively compare NP deposition behavior under steady state condition[1-9]. In this study, it was also assumed that the condition is steady state. Filtration theory claims that Brownian diffusion, interception and gravitational sedimentation are three mechanisms which affect colloid transport through saturated porous media[10-12]. To predict the single-collector efficiency (η_0) for physicochemical filtration a correlation was developed by Tufenkji and Elimelech[12] as:

$$\eta_0 = \eta_D + \eta_I + \eta_G \quad (1)$$

where η_D, η_I and η_G are transport due to diffusion, interception and gravity respectively and are calculated as:

$$\eta_0 = 2.4 A_S^{\frac{1}{3}} N_R^{-0.081} N_{Pe}^{-0.715} N_{vdw}^{0.052} + 0.55 A_S\ N_R^{1.675} N_A^{0.125} + 0.22 N_R^{-0.24} N_G^{1.11} N_{vdw}^{0.053} \quad (2)$$

where A_S is the Happel correction factor, N_R is the interception number, N_{Pe} is the Peclet number, N_{vdw} is the London-van der Waals attractive forces number, N_A is the attraction number, and N_G is the gravitational number and calculate as:

$$N_R = \frac{d_p}{d_c} \quad (3)$$

$$N_{Pe} = \frac{Ud_c}{\dfrac{kT}{6\pi\mu a_p}} \quad (4)$$

$$N_{vdw} = \frac{A}{kT} \quad (5)$$

$$N_A = \frac{A}{12\pi\mu a_p^2 U} \quad (6)$$

$$N_G = \frac{2a_p^2 g(\rho_p - \rho_f)}{9\mu U} \quad (7)$$

$$A_S = \frac{2(1-\varphi_w^5)}{2 - 3\varphi_w + 3\varphi_w^5 - 2\varphi_w^6} \quad (8)$$

where d_c is the collector diameter (150 µm), d_p is the particle diameter (255, 220, and 280 nm for Al_2O_3, TiO_2, and SiO_2 respectively), U is the fluid approach velocity (4.67×10^{-5} m/sec), A is the Hamaker constant (refer to Table S1), T is fluid absolute temperature (298.15 ^{o}K), k is the Boltzmann constant (1.3805×10^{-23} J/^{o}K), a_p is particle radius, ρ_f is the fluid density (refer to Table S2), ρ_p is the particle density (3.9, 3.8, 2.51 g/cm^3 for Al_2O_3, TiO_2, and SiO_2

respectively), μ is the absolute fluid viscosity (refer to Table S2), φ_w is porosity of porous media, and g is the gravitational acceleration (9.82 m/sec^2).

In addition, the attachment efficiency factor (α) which is a function of the physicochemical parameters of the systems, is calculated as:

$$\alpha = -\frac{2d_c}{3(1\text{-}\varphi)L\eta_0}\ln\left(\frac{\mathrm{C}}{\mathrm{Co}}\right) \tag{9}$$

where L is the length of the column, φ is porosity, and C/C_O is the normalized NP concentration acquired from the experimental breakthrough curves. The average value of C/C_O between PVs of 1.8 to 2.0 was applied in equation 10, where the initial (clean bed) phase of NP elution exists.

In addition, He *et al.*[13] proposed a correlation to calculate maximum distance (L_{max}) that colloids can travel in a saturated porous medium. They found that L_{max} is the distance where 99% of the colloids are removed from the pore solution. L_{max} can be calculated as:

$$L_{max} = -\frac{2d_c}{3(1-\varphi)\alpha\eta_0}\ln(0.01) \tag{10}$$

During evaluation of colloidal transport through saturated porous media via filtration theory, the rate of physicochemical filtration is presented by the particle deposition rate coefficient (k_d). This coefficient is defined as:

$$k_d = \frac{3\,(1-\varphi)}{2d_c\varphi}U\alpha\eta_o \tag{11}$$

where U is Darcy velocity.

Table S1. Hamaker constant values

	A_{131}	A_{132}		
		limestone	dolomite	quartz sand
Al_2O_3	5.3E-20	3.67E-20	3.67E-20	3.40E-20
TiO_2	6.0E-20	1.4E-20	1.4E-20	1.71E-20
SiO_2	8.5E-21	7.7E-21	7.7E-21	6.6E-21

Table S2. Nanofluids properties at 26°C

Fluid	Density (g/cm^3)	Viscosity (cP)	pH
Al_2O_3 nanofluid, 0.005wt%	0.9901	1.44±0.02	6.75
TiO_2 nanofluid, 0.005wt%	0.9908	1.65±0.02	6.35
SiO_2 nanofluid, 0.005wt%	0.9919	1.28±0.02	6.65

3. DLVO Theory

DLVO theory was applied to qualitatively understand the interactions between NP and NP and also between NP and the limestone grain (collector). For this aim, the total NP-NP and NP-collector interaction energies as the summation of van der Waals (VDW) and electrostatic double layer (EDL) interactions were calculated. For the calculation of the NP-NP interaction energy, it was assumed that NPs are spherical while for NP-collector interaction, it is assumed that the NP is spherical and collector is flat[14-15]. The VDW attractive interaction energies for the NP-NP (E_{VDW-NN}) and NP-collector (E_{VDW-NC}) systems are computed as:

$$E_{VDW-NN} = -\frac{A_{131}}{6}\left[\frac{2a_p^{\,2}}{D\left(4a_p + D\right)} + \frac{2a_p^{\,2}}{\left(2a_p + D\right)^2} + \ln\left(\frac{D\left(4a_p + D\right)}{\left(2a_p + D\right)^2}\right)\right] \quad (12)$$

$$E_{VDW-NC} = -\frac{A_{132} a_p}{6D}\left[1+\frac{14D}{\lambda}\right]^{-1} \tag{13}$$

where A_{131} is Hamaker constant for NP-water-NP (for Al_2O_3 NP-NP 5.3×10^{-20} *J*; for TiO_2 NP-NP 6.0×10^{-20} *J*; SiO_2 NP-NP 8.5×10^{-21} *J* were utilized[16]) and A_{132} is Hamaker constant for NP-water-collector (the values are shown in Table S1), a_p is the radii of NP aggregates, D is the separation distance, and λ is the characteristic wavelength of interaction that is assumed 100 nm.

In addition, the EDL interaction energies for the NP-NP (E_{EDL-NN}) and NP-collector (E_{EDL-NC}) systems are calculated as:

$$E_{EDL-NN} = \frac{64\pi\varepsilon_0\varepsilon_r k_B{}^2 T^2 a_p^2}{e^2 z^2} \times \tanh^2\left(\frac{ze\Psi_p}{4k_BT}\right) \times \frac{\exp(-\kappa D)}{2a+D} \tag{14}$$

$$E_{EDL-NC} = \pi\varepsilon_0\varepsilon_r a_p\left(\xi_p^2+\xi_c^2\right)\left\{\frac{2}{(\xi_p^2+\xi_c^2)}\ln\left(\frac{1+\exp(-\kappa D)}{1-\exp(-\kappa D)}\right)+\ln(1-\exp(-2\kappa D))\right\} \tag{15}$$

where k_B is the Boltzmann constant (1.3805×10^{-23} J/^{o}K), T is the absolute temperature of the system (298.15 ^{o}K), ε_0 is the permittivity of free space (8.85×10^{-12} C/V/m), ε_r is the relative dielectric constant of water (78.5), Ψ_p is the reduced potential (Ψ = ze ξ /k_BT) of NP, ξ_p and ξ_c are the electrical potentials of the NP aggregate and the collector, *e* is the electron charge (1.602×10^{-19} C), z is the valence of ion in bulk solution, and κ is the Debye-Hückel reciprocal length and is calculated as:

$$\kappa = \left(\frac{2000 e^2 N_A I}{\varepsilon_0\varepsilon_r k_B T}\right)^{0.5} \tag{16}$$

where N_A is the Avogadro's number (6.022×10^{23}) and I is the ionic strength (for DIW it is assumed $10^{-5.5}$ M[17]).

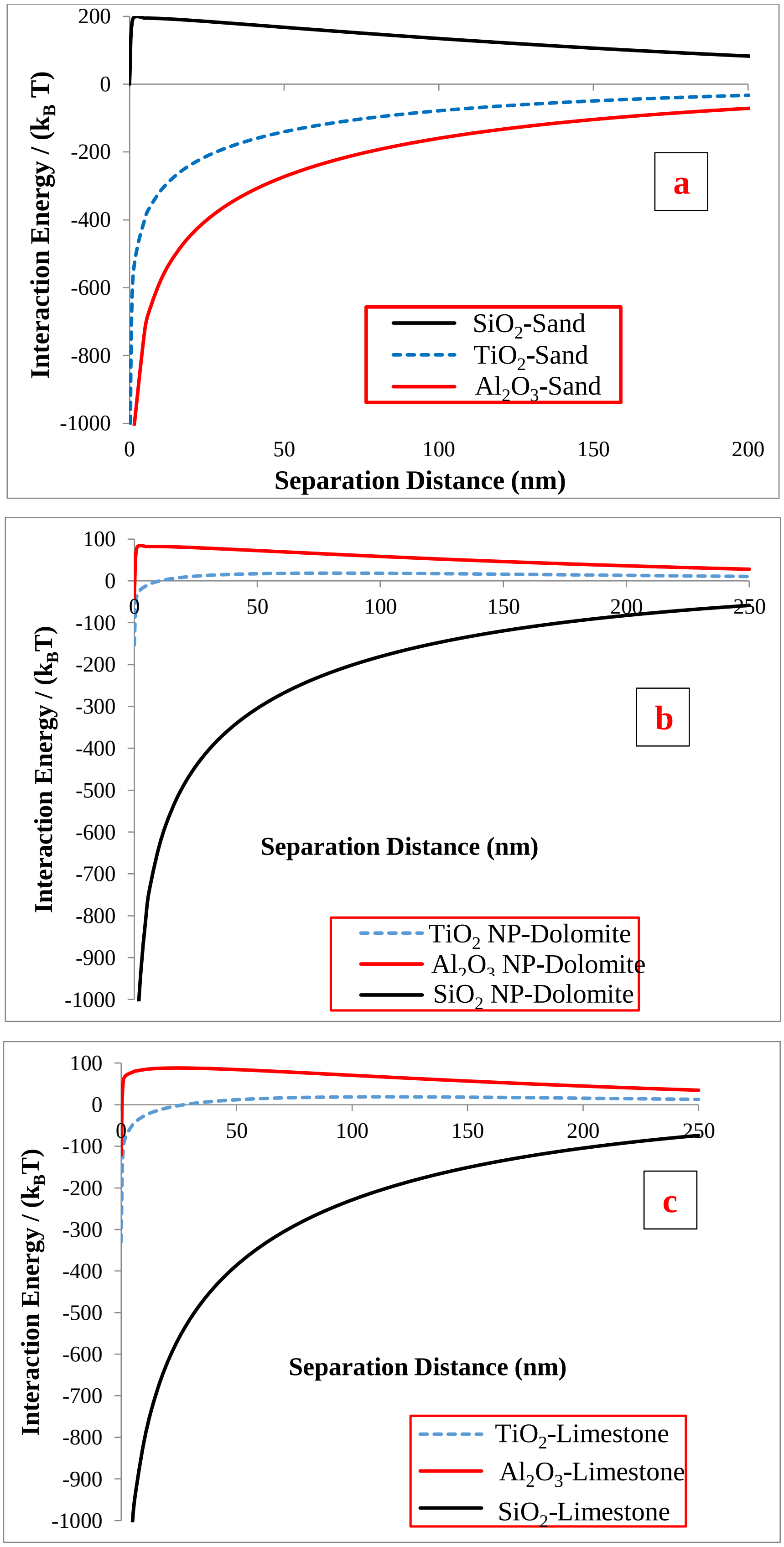

Figure S1. Interaction energy profiles between NPs-Porous media grains generated by DLVO theory, (a) NPs-quartz sand, (b) NPs-dolomite, (c) NPs-limestone.

References:

1- Dunphy Guzman, K.A., Finnegan, M.P., Banfield, J.F. Influence of Surface Potential on Aggregation and Transport of Titania Nanoparticles. *Environ. Sci. Technol*. **40**, 7688-7693 (2006).

2- Lecoanet, H.F., Bottero, J.Y., Wiesner, M.R. Laboratory Assessment of the Mobility of Nanomaterials in Porous Media. *Environ. Sci. Technol*. **38**, 5164-5169 (2004).

3- Phenrat, T., Saleh, N., Sirk, K., Tilton, R.D., Lowry, G.V. Aggregation and Sedimentation of Aqueous Nanoscale Zerovalent Iron Dispersions. Environ. Sci. Technol. **41**, 284-290 (2006).

4- French, R.A. et al. Influence of Ionic Strength, pH, and Cation Valence on Aggregation Kinetics of Titanium Dioxide Nanoparticles. *Environ. Sci. Technol*. **43**,1354-1359(2009).

5- Godinez, I.G., Darnault, C.J.G. Aggregation and transport of nano-TiO_2 in saturated porous media: Effects of pH, surfactants and flow velocity. *Water Research* **45**, 839-851 (2011).

6- Petosa, A.R., Brennan,S.J., Rajput, F., Tufenkji, N. Transport of two metal oxide nanoparticles in saturated granular porous media: Role of water chemistry and particle coating. *Water Research* **46**, 1273-1285 (2012).

7- Jiang, X., Tong, M., Lu, R., Kim, H. Transport and deposition of ZnO nanoparticles in saturated porous media. *Colloids and Surfaces A: Physicochemical and Engineering Aspects* **401**, 29-37 (2012).

8- Esfandyari Bayat, A., Junin, R., Ghadikolaei,F.D., Piroozian, A. Transport and aggregation of Al_2O_3 nanoparticles through saturated limestone under high ionic strength conditions: measurements and mechanisms. *J Nanopart Res*. **16**, 1-12 (2014).

9- Li, Z., Sahle-Demessie, E., Hassan, A.A., Sorial, G.A. Transport and deposition of CeO_2 nanoparticles in water-saturated porous media. *Water Research* **45**, 4409-4418 (2011).

10- Yao, K.M., Habibian, M.T., O'Melia, C.R. Water and waste water filtration. Concepts and applications. *Environ. Sci. Technol*. **5**, 1105-1112 (1971).

11- Elimelech, M., Jia, X., Gregory, J., Williams, R. Particle Deposition & Aggregation: Measurement, Modelling and Simulation. First ed., Butterworth-Heinemann, United States of America, (1998).

12- Tufenkji, N., Elimelech, M. Deviation from the Classical Colloid Filtration Theory in the Presence of Repulsive DLVO Interactions. *Langmuir* **20**, 10818-10828 (2004).

13- He, F., Zhang, M., Qian, T., Zhao, D. Transport of carboxymethyl cellulose stabilized iron nanoparticles in porous media: Column experiments and modeling. *J Colloid and Interface Sci*. **334**, 96-102 (2009).

14- Chen, K.L., Elimelech, M. Influence of humic acid on the aggregation kinetics of fullerene (C_{60}) nanoparticles in monovalent and divalent electrolyte solutions. *J Colloid and Interface Sci.* **309**, 126-134 (2007).

15- Chowdhury, I., Hong, Y., Honda, R.J., Walker, S.L. Mechanisms of TiO_2 nanoparticle transport in porous media: Role of solution chemistry, nanoparticle concentration, and flowrate. *J Colloid and Interface Sci*. **360**, 548-555 (2011).

16- Petosa, A.R., Jaisi, D.P., Quevedo, I.R., Elimelech, M., Tufenkji, N. Aggregation and Deposition of Engineered Nanomaterials in Aquatic Environments: Role of Physicochemical Interactions. *Environ. Sci. Technol*. **44**, 6532-6549 (2010).

17- Chen, G., Liu, X., Su, C. Transport and Retention of TiO_2 Rutile Nanoparticles in Saturated Porous Media under Low-Ionic-Strength Conditions: Measurements and Mechanisms. *Langmuir* **27**, 5393-5402 (2011).
